# Phenotypic variation of floral organs in *Malus* using frequency distribution functions

**DOI:** 10.1186/s12870-019-2155-6

**Published:** 2019-12-21

**Authors:** Ting Zhou, Junjun Fan, Mingming Zhao, Donglin Zhang, Qianhui Li, Guibin Wang, Wangxiang Zhang, Fuliang Cao

**Affiliations:** 1 College of Forestry, Nanjing Forestry University, Nanjing, 210037 China; 2 Co-Innovation Center for Sustainable Forestry in Southern China, Nanjing Forestry University, Nanjing, 210037 China; 3 Department of Horticulture, University of Georgia, Athens, GA 30602 USA; 4 Yangzhou Crabapple Horticulture Company Limited, Yangzhou, 225200 China

**Keywords:** *Malus* spp., Floral variation, Direction, Degree, Frequency distribution

## Abstract

**Background:**

Phenotypic diversity of floral organs plays an important role in plant systematic taxonomy and genetic variation studies. Previous research have focused on the direction of variation but disregarded its degree. Phenotypic variation (including directions and degrees) of 17 floral traits from wild to cultivated crabapples were explored by comparing their distributions and deviations in three different dimensions: floral organ number, size, and the shape.

**Results:**

Except for petal number, petal length / petal width, and sepal length / sepal width, the analyzed floral traits of cultivated crabapples all showed downward distributed box bodies in box plot analysis and left deviations of fitted curves in frequency distribution function analysis when compared to the wild, which revealed consistent variation directions of petaloid conversion (pistils or stamens → petals), size miniaturization (large → small), and shape narrowness (petal shape: circular → elliptic; sepal shape: triangular → lanceolate). However, only seven floral traits exhibited significant differences in box plot analysis, while all of the traits in frequency distribution function analysis were obviously offset. The variation degrees were quantitatively characterized by sizing traits > shaping traits > numbering traits and by horizontal dimensions > radial dimensions.

**Conclusions:**

Frequency distribution function analysis was more sensitive than the box plot analysis, which constructed a theoretical basis for *Malus* flower type breeding and would provide a new quantitative method for future evaluation of floral variation among different groups of angiosperms at large.

## Background

Crabapples (*Malus* spp.) are small trees and shrubs in the rose family, valued for their charming flowers, colorful small fruits (≤5 cm), and diverse growth habits. They also have an added advantage of wide environmental adaptability, facilitating their world-wide prominence as landscape and gardens focal points [1–3]. After a long period of natural selection and crossbreeding, *Malus* germplasm present a high level of diversity, with a steadily increasing number of varieties and cultivars in relation to their wild ancestors [4–7]. While nearly 1200 *Malus* taxa are recorded in Fiala’s “*Flowering Crabapple*” book, less than 5% are semi-double or double flowered. Additionally, germplasm with larger flowers are also rare, resulting in a scarcity of double-flowered and novel-typed cultivars available in today’s market [8].

Flowers are one of the most ornamental features of garden plants [9–11]. They display extremely high variation in size, color, structure, and function, which are the products of continuous remodeling to adapt to different environmental conditions and pollinators and the important foundations for germplasm innovations [12–19]. Currently, most floral variation studies have been restricted to the anatomical examinations and genetic interpretations for their development on the basis of phylogenetics and molecular genetics, combining with the ABC (DE) and the quartet models [20–28]. Moreover, variation analyses mainly occurred above the species level (at the macro level, mainly determined by paleontology and comparative morphology methods) [29–31]. Based on phenotypes and statistical principles, few studies were carried out with more intuitive estimations of floral variation below the species level (at the micro level, mostly determined by genetics, ecology, and low-level systematics methods) [30, 31]. Chu et al. (2009) summarized the main phenotypic variation of *Malus* floral organs based on intuitive experience and comparative morphology, which had been widely accepted by researchers [32–34]. These results, however, were relatively imprecise because of the subjectivity of the methods. Furthermore, these studies did focused on the direction of variation and disregarded its degree.

Using box plot and frequency distribution function analyses, phenotypic variation of floral organs from wild to cultivated crabapples were explored to: (1) determine the variation rules (including directions and degrees) governing floral changes between the two groups in three different dimensions: floral organ number, size, and the shape; (2) compare the effect of different analytical methods on generating the variation rules; and (3) provide a theoretical basis for the inheritance and improvement of *Malus* germplasm.

## Results

### Box plot analysis of phenotypic variation of floral organs between wild and cultivated crabapples

**Fig. 1:**
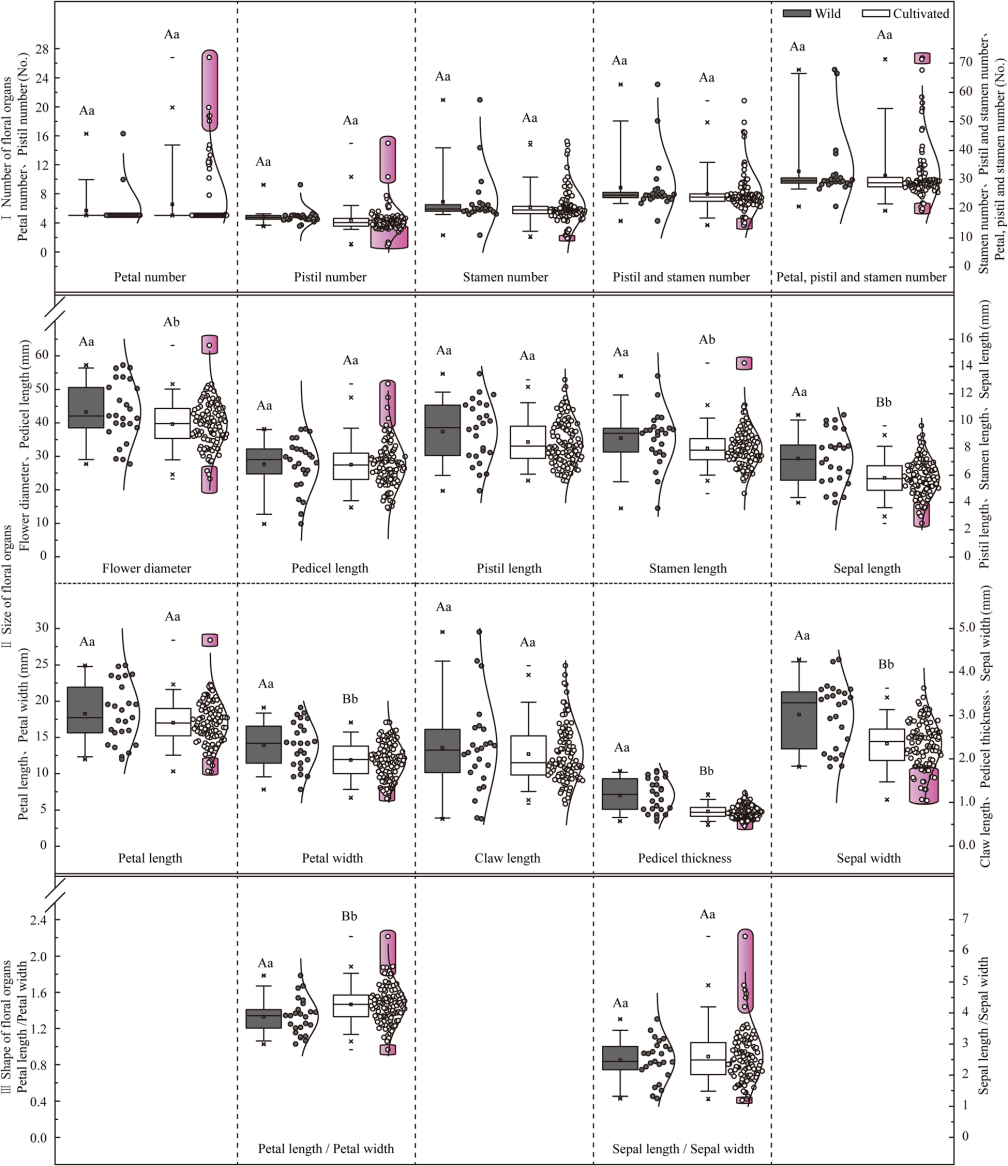
Box plots of floral phenotypic traits of wild and cultivated crabapples. The middle region of each box plot (box body) covers 50% of the individuals, and the region between the upper and the lower transverse lines covers 90% of the individuals, whereas the points outside of the box highlighted in purple represent transgressive individuals. The mean values are presented in small squares inside the box bodies. Datasets of all the wild and cultivated individuals are shown on the right of each box plots and their distributions are fitted with a line. The different lowercase letters indicate significant differences (*P* < 0.05) between the mean values of each floral traits from wild and cultivated crabapple groups, while different capital letters indicate highly significant differences (*P* < 0.01) between the mean values of each traits from these two groups. Identical letters indicate the absence of significant differences

Figure 1 shows box plots for 17 phenotypic traits reflecting *Malus* floral organ number, size, and shape. Comparative analyses of distributions and differences between the two groups were carried out, one including 25 wild crabapples and the other including 108 cultivated ones. Except for petal number, petal length / petal width, and sepal length / sepal width, all cultivated crabapples’ box bodies of the other floral traits showed downward distributions relative to the wild. Specific distributions of all the phenotypic traits were as follows:

In terms of floral organ number (Fig. 1-I), petal number, pistil number, and petal, pistil and stamen number of cultivated crabapples were distributed across a larger range that completely encompassed the distributions of wild crabapples (the proportions of transgressive individuals were 4.6, 23.1 and 6.5%, respectively). However, with regards to the stamen number and the pistil and stamen number, smaller distribution ranges were shown in the cultivated relative to the wild, although certain transgressive individuals (smaller individuals) still existed (the proportions of transgressive individuals were 2.8 and 4.6%, respectively). No significant differences were determined among these numbering traits between the two groups (*P* values were 0.2896, 0.1021, 0.4922, 0.1959, and 0.1394, respectively).

In terms of floral organ size (Fig. 1-II), larger distribution ranges were shown in flower diameter, petal length, sepal length, sepal width, and pedicel length of cultivated crabapples. Among them, distribution ranges of flower diameter and petal length of cultivated crabapples completely encompassed the ranges of wild ones (the proportions of transgressive individuals were both 5.6%). Downward distribution trends were presented in sepal length and sepal width, with smaller values in cultivated crabapples than the wild; whereas for the pedicel length, the upward distribution trend was presented together with higher values (the proportions of transgressive individuals were 9.3, 15.7, and 5.6%, respectively). On the contrary, sizing traits of petal width, claw length, pistil length, stamen length and pedicel thickness of cultivated crabapples were distributed across a smaller range. Distribution ranges of claw length and pistil length of cultivated crabapples were completely encompassed by those of the wild. And downward distribution trends were presented in petal width and pedicel thickness, with smaller values in cultivated crabapples than the wild; whereas the upward distribution trend was presented in stamen length together with higher values (the proportions of transgressive individuals were 5.6, 5.6, and 0.9%, respectively). Except for pedicel length (*P* = 0.9660), pistil length (*P* = 0.0567), petal length (*P* = 0.0783), and claw length (*P* = 0.4040), the other six sizing traits of flower diameter, petal width, sepal length, sepal width, stamen length, and pedicel thickness, all showed significant differences between the two groups (*P* values were 0.0244, 0.0005, 0.0001, 0.0001, 0.0237, and 0.0001, respectively).

In terms of floral organ shape (Fig. 1-III), petal length / petal width and sepal length / sepal width of cultivated crabapples were both distributed across a larger range that completely encompassed the distribution ranges of those in wild crabapples (the proportions of transgressive individuals were 8.3 and 7.4%, respectively). Significant variation was presented in petal length / petal width between wild and cultivated groups (*P* = 0.0030); however, differences in sepal length / sepal width did not reach the significant level (*P* = 0.5298).

### Frequency distribution function analysis of phenotypic variation of floral organs between wild and cultivated crabapples

**Fig. 2:**
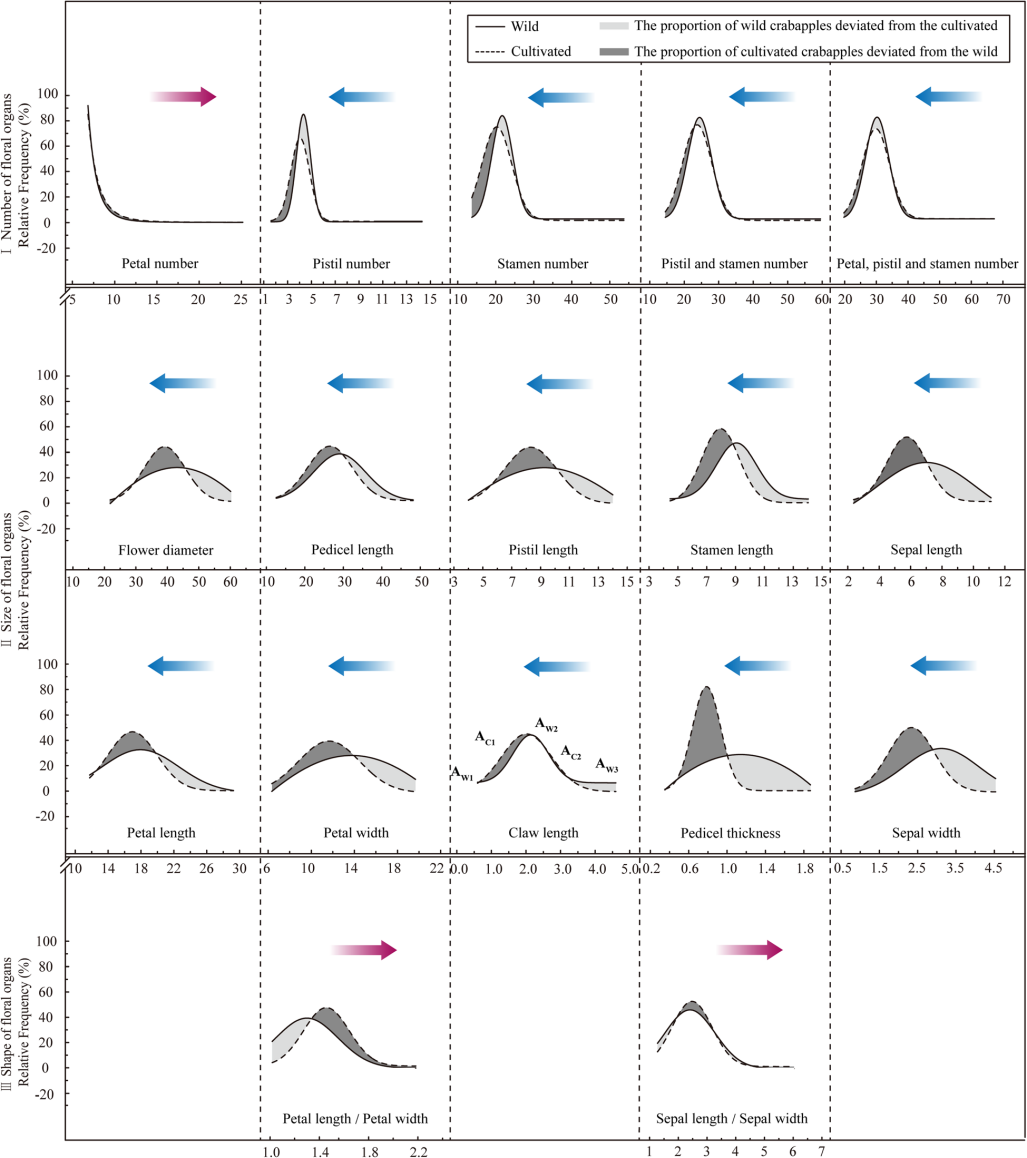
Frequency distributions of floral phenotypic traits of wild and cultivated crabapples. The areas filled with light gray indicate the probabilities of wild crabapples deviating from the cultivated, while dark gray represents the probabilities of cultivated crabapples deviating from the wild. Variation directions from wild to cultivated crabapples were presented in purple (right deviations) and blue (left deviations) arrows. The right deviations mean that compared with wild crabapples, floral traits of cultivated exhibited increasing trends with larger values, whereas the left deviations mean the opposite

For a clearer analysis of floral variation from wild to cultivated crabapples, frequency distribution functions of all the 17 above-mentioned phenotypic traits were fitted (Fig. 2). Except for petal number, which followed a power function distribution (R^2^ = 0.9931–0.9972), all the other floral traits followed the normal distribution (R^2^ = 0.8625–0.9991) (Table 1).

**Table 1:** Frequency distribution functions and variation degrees of *Malus* floral traits

Phenotypic traits of floral organs		Frequency distribution functions		Degrees of phenotypic variation				
		Wild crabapples	Cultivated crabapples	MD	Order	MP	Order	Rank
Numbering traits	Petal number	y = 102154000 ⋅ *x*^(−7.243)^(R^2^ = 0.9972)	y = 11002600 ⋅ *x*^(−6.124)^(R^2^ = 0.9931)	/	/	0.0520	17	III
	Pistil number	$\mathrm{y}=0.571+84.548\cdot {e}^{-1.411{\left(x-4.328\right)}^2}$(R^2^ = 0.9967)	$\mathrm{y}=0.963+65.342\cdot {e}^{-0.825{\left(x-4.052\right)}^2}$(R^2^ = 0.9965)	0.0277	15	0.1967	9	
	Stamen number	$\mathrm{y}=2.667+81.333\cdot {e}^{-0.064{\left(x-21.700\right)}^2}$(R^2^ = 0.9951)	$\mathrm{y}=1.541+73.573\cdot {e}^{-0.033{\left(x-20.273\right)}^2}$(R^2^ = 0.9973)	0.0439	13	0.1736	11	
	Pistil and stamen number	$\mathrm{y}=2.667+80.144\cdot {e}^{-0.042{\left(x-24.427\right)}^2}$(R^2^ = 0.9945)	$\mathrm{y}=1.541+75.431\cdot {e}^{-0.028{\left(x-23.737\right)}^2}$(R^2^ = 0.9987)	0.0389	14	0.0941	14	
	Petal, pistil and stamen number	$\mathrm{y}=2.667+80.144\cdot {e}^{-0.037{\left(x-30.228\right)}^2}$(R^2^ = 0.9781)	$\mathrm{y}=2.776+71.000\cdot {e}^{-0.027{\left(x-29.825\right)}^2}$(R^2^ = 0.9989)	0.0261	16	0.0690	15	
Sizing traits	Flower diameter	$\mathrm{y}=-134.198+162.225\cdot {e}^{-0.004{\left(x-43.110\right)}^2}$(R^2^ = 0.9664)	$\mathrm{y}=1.154+43.064\cdot {e}^{-0.011{\left(x-39.146\right)}^2}$(R^2^ = 0.9982)	−2.7975	2	0.2280	7	I
	Pedicel length	$\mathrm{y}=1.897+36.917\cdot {e}^{-0.011{\left(x-29.016\right)}^2}$(R^2^ = 0.9277)	$\mathrm{y}=1.998+42.694\cdot {e}^{-0.015{\left(x-26.524\right)}^2}$(R^2^ = 0.9924)	−0.2041	10	0.1487	12	
	Pistil length	$\mathrm{y}=-65.441+93.426\cdot {e}^{-0.012{\left(x-9.265\right)}^2}$(R^2^ = 0.8625)	$\mathrm{y}=-0.335+44.185\cdot {e}^{-0.147{\left(x-8.309\right)}^2}$(R^2^ = 0.9802)	−1.9783	4	0.2233	8	
	Stamen length	$\mathrm{y}=3.251+44.014\cdot {e}^{-0.256{\left(x-9.065\right)}^2}$(R^2^ = 0.9695)	$\mathrm{y}=0.405+58.096\cdot {e}^{-0.303{\left(x-7.956\right)}^2}$(R^2^ = 0.9988)	−0.3078	9	0.3105	4	
	Sepal length	$\mathrm{y}=-17.100+49.152\cdot {e}^{-0.049{\left(x-7.007\right)}^2}$(R^2^ = 0.9935)	$\mathrm{y}=1.279+50.647\cdot {e}^{-0.309{\left(x-5.765\right)}^2}$(R^2^ = 0.9961)	−1.1178	5	0.3121	3	
	Petal length	$\mathrm{y}=-1.393+33.994\cdot {e}^{-0.023{\left(x-17.938\right)}^2}$(R^2^ = 0.9911)	$\mathrm{y}=0.222+46.496\cdot {e}^{-0.056{\left(x-16.959\right)}^2}$(R^2^ = 0.9980)	−0.4758	7	0.1835	10	
	Petal width	$\mathrm{y}=-134.198+162.225\cdot {e}^{-0.004{\left(x-13.734\right)}^2}$(R^2^ = 0.9664)	$\mathrm{y}=-1.350+40.760\cdot {e}^{-0.062{\left(x-11.650\right)}^2}$(R^2^ = 0.9944)	−2.5597	3	0.2651	5	
	Claw length	$\mathrm{y}=6.466+37.808\cdot {e}^{-1.875{\left(x-2.139\right)}^2}$(R^2^ = 0.9749)	$\mathrm{y}=-0.414+45.463\cdot {e}^{-0.958{\left(x-2.018\right)}^2}$(R^2^ = 0.9211)	0.1417	12	0.1008	13	
	Pedicel thickness	$\mathrm{y}=-963.219+992.038\cdot {e}^{-0.046{\left(x-1.139\right)}^2}$(R^2^ = 0.8755)	$\mathrm{y}=0.311+81.904\cdot {e}^{-22.977{\left(x-0.786\right)}^2}$(R^2^ = 0.9987)	−13.2426	1	0.5499	1	
	Sepal width	$\mathrm{y}=-3.203+36.806\cdot {e}^{-0.519{\left(x-3.118\right)}^2}$(R^2^ = 0.9661)	$\mathrm{y}=-0.736+50.877\cdot {e}^{-1.385{\left(x-2.340\right)}^2}$(R^2^ = 0.9980)	−0.9277	6	0.4135	2	
Shaping traits	Petal length / petal width	$\mathrm{y}=-0.287+39.567\cdot {e}^{-7.874{\left(x-1.299\right)}^2}$(R^2^ = 0.9738)	$\mathrm{y}=1.356+46.233\cdot {e}^{-14.595{\left(x-1.457\right)}^2}$(R^2^ = 0.9991)	0.4330	8	0.2438	6	II
	Sepal length /sepal width	$\mathrm{y}=-0.689+46.502\cdot {e}^{-0.658{\left(x-2.405\right)}^2}$(R^2^ = 0.9900)	$\mathrm{y}=0.958+51.566\cdot {e}^{-1.032{\left(x-2.475\right)}^2}$(R^2^ = 0.9966)	0.1557	11	0.0686	16	

Note: The order from 1...17 and the rank from I...III at the end of the table indicate a gradual reduction in the degree of phenotypic variation

From wild crabapples to the cultivated, the power distribution function of petal number showed a right deviation (increasing trend), while normal distribution functions of the other numbering traits (pistil number, stamen number, pistil and stamen number, and petal, pistil and stamen number) showed the opposite, which indicated that additional petals in cultivated crabapples might arise from petaloid conversions of pistils or stamens during the doubling processes (Fig. 2-I). Consistent trends of left deviations (decreasing trends) were shown in all the sizing traits (Fig. 2-II), while normal distribution functions of the shaping traits both showed the contrary (Fig. 2-III).

To quantitatively express the degree of phenotypic variation of floral organs between wild and cultivated crabapples, two characteristic parameters; namely, misregistration distance (MD) and misregistration probability (MP), were calculated (Table 1). Significant positive correlation was shown between MD and MP (*r* = 0.7880, *P* = 0.0000), confirming the validity of these two parameters. Overall, the sizing traits of floral organs showed the highest degree of variation, followed by the shaping and numbering traits. Phenotypic variation occurred higher in the horizontal dimension (pedicel thickness, petal width, and sepal width) than that in the radial dimension (pedicel length, petal length, and sepal length).

## Discussion

### Additional petals in cultivated crabapples arose from petaloid conversions of pistils or stamens during the long period of natural selection and crossbreeding

“Double” refers to flowers with more than one petal whorl or additional petals [35]. This phenomenon can be produced by either neoheterotopy or homoheterotopy. Neoheterotopy refers to an increase in the number of petal whorls in sympetalous flowers [36, 37], whereas homoheterotopy refers to the petaloid conversion of pistils and stamens [38–44] or bracts and sepals to construct additional petals [45–48]. Chu (2009) proposed that additional petals in cultivated crabapples were petaloid stamens [32], which had been acknowledged by many researchers. In this study, however, results were different. With an increasing trend of petal number, pistil number and stamen number of cultivated crabapples both decreased in a relatively equivalent degree (MP _pistil number_ = 0.1967, MP _stamen number_ = 0.1736; MD _pistil number_ = 0.0277, MD _stamen number_ = 0.0439), suggesting that multiple petals might be derived from pistils or stamens. The incomplete agreement between these two viewpoints described above could be mainly due to the differences in materials and methods used. Chu’s study was mainly based on scattered discoveries, while a total of 133 *Malus* taxa were investigated in the present study, including 25 wild crabapples (accounting for 71.4% of total wild species recorded in the flora of China) and 108 cultivated ones (accounting for more than 50.0% of total cultivars that could be found in nurseries). Among the cultivated crabapples were 18 semi-double or double types, accounting for approximately 69.2% of the documented double types in Fiala’s (1994) “Flowering Crabapple” book [8]. The representativeness of these research materials, to a certain extent, determined the reliability of this study results. Regarding the research methods, a comparative morphological method with a certain subjectivity was applied in Chu’s study, which resulted in less precise conclusions. Instead, quantitative assessment based on statistical principles was adopted in the present study, which revealed the origin of the doubling phenomenon in cultivated crabapples more clearly and provided a more precise theoretical basis for *Malus* double-typed cultivars breeding.

### Non-additive effects contributed to the inhomogeneous miniaturization of floral size in cultivated crabapples

Compared to the wild crabapples, 10 sizing traits of the cultivated exhibited a consistent trend of miniaturization with inhomogeneous variation degrees. Decrease in petal length resulted in smaller flower diameter (*P*_petal length_ < *P*_claw length_), while larger degree of variation in the horizontal rather than in the radial dimension led to smaller and narrower floral shapes (MP _petal length_ = 0.1835, MP _petal width_ = 0.2651, MP _sepal length_ = 0.3121, and MP _sepal width_ = 0.4135). To account for this miniaturization, non-additive effects (including dominant and epistasis effects) were proposed [49, 50]. Although *Malus* taxa originate from wild species, their genotypes are highly heterozygous after long-term natural selection and crossbreeding. According to the dominance hypothesis, dominant alleles are favored over recessive alleles for the growth and development of individuals [51]. Self-crossing or inbreeding of these heterozygous individuals will therefore increase the production of homozygotes in hybrids and expose the harmful traits represented by recessive genes, which will lead to the hybrid depression and significantly reduce the probability of heterosis [52–56]. Additionally, Li (2007) proposed that in F_1_ hybrid of *Gerbera jamesonii*, the average values of flower diameter, pedicel length and ray floret were significantly smaller than those of their parents, which may be due to the one-way selection in the process of breeding and the large amount of non-additive effects reserved by asexual reproduction on the preservation of selected cultivars. Once sexual reproduction occurs, the possibility of heterosis may be reduced, resulting in a decrease in average values of hybrid group’s traits [57]. Do this one-way selection, as well as the different fixation and heredity of non-additive effects produced by asexual and sexual reproduction, also exist in the previous breeding process of *Malus* taxa, which can lead to the depression of all sizing traits in the progeny? These questions deserve further exploration. Nevertheless, transgressive individuals with higher values still existed in sizing traits of flower diameter, petal length, stamen length, and pedicel length (the proportions of transgressive individuals were 5.6, 5.6, 0.9, and 5.6%, respectively), which would provide a possibility for further innovations of *Malus* germplasm, such as large-flowered cultivars.

### Frequency distribution function analysis was more sensitive than box plot analysis, revealing clearer phenotypic variation of *Malus* flowers

Plants can evolve on both the macro and micro levels. Evolution at the level of genera and higher taxonomic levels (interfamily, etc.) can be regarded as macro-evolution, reflecting the origins and phylogenetic processes of large-scale alterations in plant taxa during long geological ages. In contrast, evolution within genera (inter-species and inter-cultivar) can be regarded as micro-evolution, reflecting the evolutionary processes of small-scale alterations in plant taxa during shorter time periods [30]. The significant differences at the level of observation between macro- and micro-evolution determine the differences in their respective research methods. The former is mainly using methods of paleontology and comparative morphology, while the latter is mainly using methods of genetics, ecology and low-level systematics [31]. In this study, phenotypic variation of floral organs between wild and cultivated crabapples represents the micro level. However, previous researchers mainly depended on intuitive experience and adopted the comparative morphological method for analysis [32–34]. This method can only roughly reveal the variation directions because of its highly subjective, leading to less reliable and imprecise results. To reveal the variation rules more objectively, accurately and thoroughly, two methods of box plot analysis (method I) and frequency distribution function analysis (method II), were applied in the present study. The variation directions revealed by both methods were consistent. However, only seven phenotypic traits; namely, flower diameter, stamen length, sepal length, petal width, pedicel thickness, sepal width, and petal length / petal width, exhibited significant differences in method I, whereas in method II, frequency distribution functions of all floral traits were obviously offset. Thus, method II was more sensitive than method I. Method I reflected the relationship between the (arithmetic) mean values that indicated the differences of two groups. These mean values, however, concealed the overall alterations. Method II reflected not only the differences in the distribution centers of two groups (mathematical expectation, *Δμ*) but also the misregistration of the probability distribution (*Δσ*). Both the misregistration distance (MD) and misregistration probability (MP), which are quantitative indices for exploring phenotypic variation (including directions and degrees) between different groups, could therefore be calculated.

## Conclusions

Phenotypic variation of floral organs between wild and cultivated crabapples were mainly characterized as petaloid conversion, size miniaturization, and shape narrowness. Traits reflecting floral organ size reflected the highest degree of variation, followed by shaping and numbering traits. Higher degree of phenotypic variation occurred in the horizontal dimension, rather than in the radial dimension. Frequency distribution function analysis revealed clearer variation rules of floral organs in *Malus* than box plot analysis, which constructed a theoretical basis for *Malus* flower type breeding and would provide a new quantitative method for future evaluation of phenotypic variation among different groups in angiosperms at large.

## Methods

### Experimental site overview

The experimental site is situated at 32°42′N latitude and 119°55′E longitude. It has a northern subtropical monsoon climate with four distinctive seasons, 16.5 °C annual average temperature, 800–1000 mm annual precipitation and a 251 d frost-free period. The soil type is sandy loam with pH 7.2 and fertile soil layers. The terrain is flat with a 1.5 m groundwater level and good irrigation and drainage conditions.

### Plant materials

A total of 133 *Malus* taxa (including 25 wild and 108 cultivated crabapples) were collected from the national repository of *Malus* spp. germplasm (Yangzhou City, Jiangsu Province, China) (Table 2). All *Malus* trees were between seven and ten years old, which enabled them to enter the full bloom phase. Thirty individuals of each cultivar were planted in a row at 2 m apart with 3 m between rows.

**Table 2:** The list of *Malus* taxa collected from the national repository of *Malus* spp. germplasm (Yangzhou City, Jiangsu Province, China)

No. Wild crabapples	No. Cultivated crabapples			
1 *Malus angustifolia*	26 *M.* ‘Abundance’	53 *M.* ‘Golden Raindrop’	80 *M.* ‘May’s Delight’	107 *M.* ‘Royal Gem’
2 *M. baccata*	27 *M.* ‘Adams’	54 *M.* ‘Gorgeous’	81 *M.* ‘Molten Lava’	108 *M.* ‘Royal Raindrop’
3 *M. domestica* var.*binzi*	28 *M.* ‘Adirondack’	55 *M.* ‘Guard’	82 *M.* ‘Neville Copeman’	109 *M.* ‘Royalty’
4 *M. floribunda*	29 *M.* ‘Almey’	56 *M. halliana* ‘Pink Double’	83 *M.* ‘Perfect Purple’	110 *M.* ‘Selkirk’
5 *M. fusca*	30 *M.* ‘Ballet’	57 *M. halliana* ‘Pink Double NFU’	84 *M.* ‘Pink Princess’	111 *M.* ‘Sentinel’
6 *M. halliana*	31 *M.* ‘Black Jade’	58 *M. halliana* ‘Pink Pillar’	85 *M.* ‘Pink Spires’	112 *M.* ‘Shelley’
7 *M. hupehensis*	32 *M.* ‘Brandywine’	59 *M. halliana* ‘Waxy’	86 *M.* ‘Praire Rose’	113 *M.* ‘Show Time’
8 *M. ioensis*	33 *M.* ‘Bride’	60 *M.* ‘Harvest Gold’	87 *M.* ‘Prairifire’	114 *M.* ‘Sieboldii NFU’
9 *M. kirghisorum*	34 *M.* ‘Butterball’	61 *M.* ‘Hillier’	88 *M.* ‘Professor Sprenger’	115 *M.* ‘Snow Winter’
10 *M. mandshurica*	35 *M.* ‘Candymint’	62 *M.* ‘Hopa’	89 *M.* ‘Profusion’	116 *M.* ‘Snowdrift’
11 *M. micromalus*	36 *M.* ‘Cardinal’	63 *M.* ‘Hydrangea’	90 *M.* ‘Purple Gem’	117 *M.* ‘Spring Glory’
12 *M. orientalis*	37 *M.* ‘Centurion’	64 *M.* ‘Indian Magic’	91 *M.* ‘Purple Pendula’	118 *M.* ‘Spring Sensation’
13 *M. platycarpa*	38 *M.* ‘Cinderella’	65 *M.* ‘Indian Summer’	92 *M.* ‘Purple Prince’	119 *M.* ‘Spring Snow’
14 *M. prunifolia*	39 *M.* ‘Coccinella’	66 *M.* ‘Irene’	93 *M.* ‘Purple Spring’	120 *M.* ‘Strawberry Jelly’
15 *M. rockii*	40 *M.* ‘Coralburst’	67 *M.* ‘John Downie’	94 *M.* ‘Radiant’	121 *M.* ‘Sugar Tyme’
16 *M. sargentii*	41 *M.* ‘Darwin’	68 *M.* ‘Kelsey’	95 *M.* ‘Rainbow’	122 *M.* ‘Superstar’
17 *M. sieversii*	42 *M.* ‘David’	69 *M.* ‘King Arthur’	96 *M.* ‘Red Baron’	123 *M.* ‘Sweet Sugartyme’
18 *M. sieversii subsp. xinjinensis*	43 *M.* ‘Diamond’	70 *M.* ‘Klehm’s Improved Bechtel’	97 *M.* ‘Red Great’	124 *M.* ‘Thunderchild’
19 *M. sikkimensis*	44 *M.* ‘Dolgo’	71 *M.* ‘Lancelot’	98 *M.* ‘Red Jade’	125 *M.* ‘Tina’
20 *M. spectabilis*	45 *M.* ‘Donald Wyman’	72 *M.* ‘Lemoinei’	99 *M.* ‘Red Jewel’	126 *M.* ‘Van Eseltine’
21 *M. sylvestris*	46 *M.* ‘Eleyi’	73 *M.* ‘Lisa’	100 *M.* ‘Red Nessy’	127 *M.* ‘Velvet Pillar’
22 *M. toringoides*	47 *M.* ‘Everest’	74 *M.* ‘Liset’	101 *M.* ‘Red Sentinel’	128 *M.* ‘Weeping Madonna’
23 *M. tschonoskii*	48 *M.* ‘Fairytail Gold’	75 *M.* ‘Lollipop’	102 *M.* ‘Red Splendor’	129 *M.* ‘White Cascade’
24 *M. turkmenorum*	49 *M.* ‘Firebird’	76 *M.* ‘Louisa’	103 *M.* ‘Regal’	130 *M.* ‘Winter Gold’
25 *M. xiaojinensis*	50 *M.* ‘Flame’	77 *M.* ‘Louisa Contort’	104 *M.* ‘Robinson’	131 *M.* ‘Winter Red’
	51 *M.* ‘Furong’	78 *M.* ‘Makamik’	105 *M.* ‘Roger’s Selection’	132 *M.* ‘Yellow Jade’
	52 *M.* ‘Golden Hornet’	79 *M.* ‘Mary Potter’	106 *M.* ‘Rudolph’	133 *M.* × *zumi* ‘Calocarpa’

### Test methods

The experiment was carried out in Spring 2017 (March–April). Ten plants of each cultivar were randomly selected. Three typical, standard and consistent full-bloom flowers of each plant were collected from the middle of the tree and the branch toward the sunny side, yielding 30 flowers in total. Then, all flowers were immediately loaded into a cooler and taken to the laboratory for further use.

**Fig. 3:**
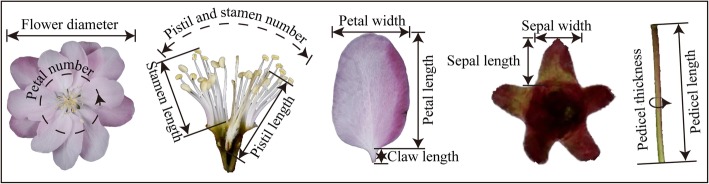
Schematic diagram of the tests on phenotypic traits of floral organs. The three numbering traits of petal number, pistil number, and stamen number in *Malus* spp. were counted for the average of 30 representative flowers. And the ten sizing traits were measured with samples straightened and pressed flat. Phenotypic traits of the others (pistil and stamen number, petal, pistil and stamen number, petal length to width, and sepal length to width) were calculated

Seventeen phenotypic traits of *Malus* floral organs were evaluated, including five numbering, ten sizing, and two shaping traits (Table 3), with samples straightened and pressed flat (Fig. 3). Thirty replicates were measured for each trait.

**Table 3:** Phenotypic traits of floral organs used in this study

Type of the traits	No.	Trait descriptor	Remarks
Numbering traits	1	Petal number	Counted
	2	Pistil number	Counted
	3	Stamen number	Counted
	4	Pistil and stamen number	Calculated
	5	Petal, pistil and stamen number	Calculated
Sizing traits	6	Flower diameter	Measured in mm
	7	Petal length	Measured in mm
	8	Petal width	Measured in mm
	9	Claw length	Measured in mm
	10	Pistil length	Measured in mm
	11	Stamen length	Measured in mm
	12	Sepal length	Measured in mm
	13	Sepal width	Measured in mm
	14	Pedicel length	Measured in mm
	15	Pedicel thickness	Measured in mm
Shaping traits	16	Petal length / Petal width	Calculated
	17	Sepal length / Sepal width	Calculated

### Data analysis

#### Box plot analysis and one-way ANOVA

Origin 9.0, DPS 7.0, and Adobe Illustrator CS5 software were used. The box plots were drawn such that the middle section (box body) covered 50% of the observation area, the section between the upper and the lower transverse lines covered 90% of the observation area, and values outside of the box were outliers. The box bodies, mean values, and outlier values were the focus of this study. Box bodies were used to explore the variation directions of each floral traits from wild to cultivated crabapples by comparing their relative positions (downward distribution with smaller values or upward distribution with higher values), whereas mean values were served to determine the significance of differences between the two groups using Duncan multiple-comparison test (*P* values of < 0.05 and < 0.01 were considered significant and highly significant, respectively), and the outliers were applied to reflect transgressive information, which would be of importance for *Malus* germplasm innovations.

#### Frequency distribution function analysis

Origin 9.0, Mathematica 9.0.1, and Adobe Illustrator CS5 software were used. Frequency distribution functions of 17 phenotypic traits of floral organs were fitted based on 6–10 frequency bins and variation rules (including directions and degrees) were explored. Except for petal number, which followed a power function distribution (y = *ax*^*b*^), all the other floral traits followed the normal function distribution $\left(y=A+B\cdot {e}^{\frac{C{\left(x-\mu \right)}^2}{\sigma^2}}\ \right)$. Parameters of misregistration distance (MD) and misregistration probability (MP) were constructed, aiming at quantitatively expressing the degrees of phenotypic variation of floral organs between wild and cultivated crabapples:

- The misregistration distance (MD) between two groups was calculated according to the characteristic parameters of the normal distribution function as follows:

$$ MD=\frac{\varDelta \mu \pm \varDelta \sigma}{R_{90}}=\frac{\left({\mu}_C-{\mu}_W\right)\pm \left({\sigma}_C-{\sigma}_W\right)}{R_{90}} $$

Where *μ*_*C*_ and *μ*_*W*_ are mathematical expectations of the random variables of cultivated and wild crabapples, respectively, that follow a normal distribution, and *σ*_*C*_ and *σ*_*W*_ are standard deviations of the random variables of the two groups, respectively, that follow a normal distribution. *R*_90_ is the range representing 90% of the observation area of cultivated crabapple's box plot, which reduces the interference of the 10% of individuals belonging to the outliers. *R*_90_ can be used as a dividend to standardize the data. In the formula, the sign ‘ ± ’ depends on the product of *Δμ* and *Δσ*. It is ‘−’ when the product is positive and ‘+’ when the product is negative.

b. The misregistration probability (MP) was calculated according to the misregistration area of the probability distribution function curve as follows:

$$ MP\left(\%\right)=\frac{A_{W1}+{A}_{W2}+{A}_{W3}}{2}+\frac{A_{C1}+{A}_{C2}}{2} $$

where *A*_*W*1_, *A*_*W*2_ and *A*_*W*3_are the probabilities of the area that were formed by the misregistration between wild and cultivated crabapples relative to the total area formed by the X-axis and the curve of the probability distribution function of the wild crabapples; and *A*_*C*1_ and *A*_*C*2_ are the probabilities of the area that were formed by the misregistration between cultivated and wild crabapples relative to the total area formed by the X-axis and the curve of the probability distribution function of the cultivated crabapples.

## Data Availability

A total of 133 *Malus* taxa (including 25 wild and 108 cultivated crabapples) were collected from the national repository of *Malus* spp. germplasm (Yangzhou City, Jiangsu Province, China). The datasets used and analyzed during the current study could be available from the corresponding author on request.

## Abbreviations

MD: Misregistration distance
MP: Misregistration probability
